# The Molecular Structure and Role of Humanin in Neural and Skeletal Diseases, and in Tissue Regeneration

**DOI:** 10.3389/fcell.2022.823354

**Published:** 2022-03-16

**Authors:** Sipin Zhu, Xiaoyong Hu, Samuel Bennett, Jiake Xu, Yuliang Mai

**Affiliations:** ^1^ Department of Orthopaedics, The Second Affiliated Hospital and Yuying Children’s Hospital of Wenzhou Medical University, Wenzhou, China; ^2^ Guangdong Provincial Key Laboratory of Industrial Surfactant, Guangdong Research Institute of Petrochemical and Fine Chemical Engineering, Guangdong Academy of Sciences, Guangzhou, China; ^3^ Division of Regenerative Biology, School of Biomedical Sciences, University of Western Australia, Perth, WA, Australia

**Keywords:** humanin, mechanisms, diseases, cell survival, neuron, skeletal, tissue regeneration

## Abstract

Humanin (HN) belongs to a member of mitochondrial-derived peptides (MDPs) which are encoded by mitochondrial genes. HN shares sequence homology with thirteen HN-like proteins, named MTRNR2L1 to MTRNR2L13, which encompass 24–28 amino acid residues in length. HN mediates mitochondrial status and cell survival by acting *via* an intracellular mechanism, or as a secreted factor via extracellular signals. Intracellularly, it binds Bcl2-associated X protein (BAX), Bim and tBid, and IGFBP3 to inhibit caspase activity and cell apoptosis. When released from cells as a secreted peptide, HN interacts with G protein-coupled formyl peptide receptor-like 1 (FPRL1/2) to mediate apoptosis signal-regulating kinase (ASK) and c-Jun N-terminal kinase (JNK) signalling pathways. Additionally, it interacts with CNTFR-α/gp130/WSX-1 trimeric receptors to induce JAK2/STA3 signalling cascades. HN also binds soluble extracellular proteins such as VSTM2L and IGFBP3 to modulate cytoprotection. It is reported that HN plays a role in neuronal disorders such as Alzheimer’s disease, as well as in diabetes mellitus, infertility, and cardiac diseases. Its roles in the skeletal system are emerging, where it appears to be involved with the regulation of osteoclasts, osteoblasts, and chondrocytes. Understanding the molecular structure and role of HN in neural and skeletal diseases is vital to the application of HN in tissue regeneration.

## 1 Introduction

The humanin (HN) protein is encoded by the *MT-RNR2* gene located within the mitochondrial genome, and was first shown to exert a neuroprotective effect, particularly in response to conditions of stress (Hashimoto et al., 2001a; Hashimoto et al., 2001b; Caricasole et al., 2002; Tajima et al., 2002; Niikura et al., 2003). HN also regulates various cell types and tissues, such as cardiovascular tissue (Widmer et al., 2013; Marleau et al., 2014; Charununtakorn et al., 2016), skeletal muscle (Gidlund et al., 2016; Lee et al., 2016), and testis (Moretti et al., 2010; Jia et al., 2013). HN appears to be implicated in a diverse range of diseases, including Alzheimer’s disease (AD), diabetes mellitus, infertility, and cardiac conditions (Mahboobi et al., 2014; Hazafa et al., 2021).

Recently using a *C. elegans* model of HN overexpression, HN was reported to increase lifespan via the regulation of daf-16/Foxo, a transcription factor involved in aging and longevity (Yen et al., 2020). HN levels declined with age and in Alzheimer’s disease, which is further suggestive of a positive role of HN for the lifespan (Yen et al., 2020). Interestingly, HN -induced autophagy plays a part in HN-induced lifespan extension in *C. elegans* (Kim et al., 2021). In addition, it was revealed that HN-induced autophagy could reduce the accumulation of harmful misfolded proteins in skeletal muscle of mice, suggestive of a role in protecting age-related diseases with degeneration (Kim et al., 2020; Kim et al., 2021). Consistently, in age-associated metabolic disorders, HN-related peptides were found to resist regulate retrograde signaling from mitochondria to endoplasmic reticulum during metabolic stress (Merry et al., 2020).

HN is widely distributed in several bodily tissues and plays roles in pathophysiology in various types of tissues and cells. Research into the roles of HN in neural and skeletal diseases is currently progressing with new insights. In this review, we first survey the molecular structure, expression and signalling of HN, and then discuss the roles of HN with a focus of recent highlights on neural and skeletal diseases, and in tissue regeneration. Understanding the tissue specific mechanisms of HN action will be vitally important for the design of personalized and relevant therapeutic applications.

## 2 Molecular Structure and Expression of HN

**FIGURE 1:**
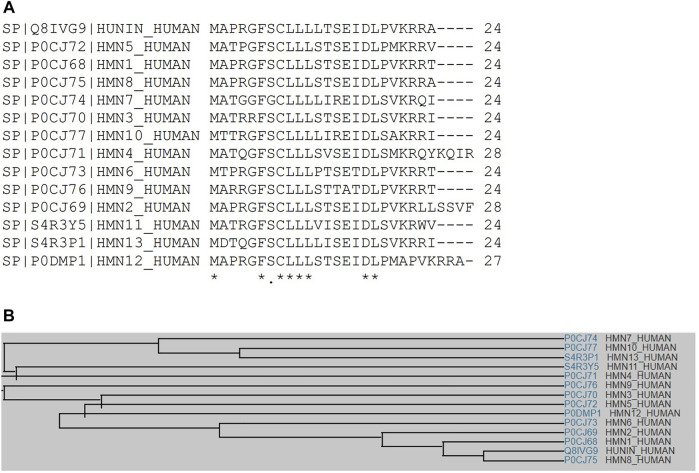
**(A)** Multiple sequence alignment analyses show that HN shares amino acid sequence identity and similarity with other HN-like proteins, MTRNR2L1 to MTRNR2L13. **(B)** A family tree of HN proteins and HN-like proteins is presented.

**FIGURE 2:**
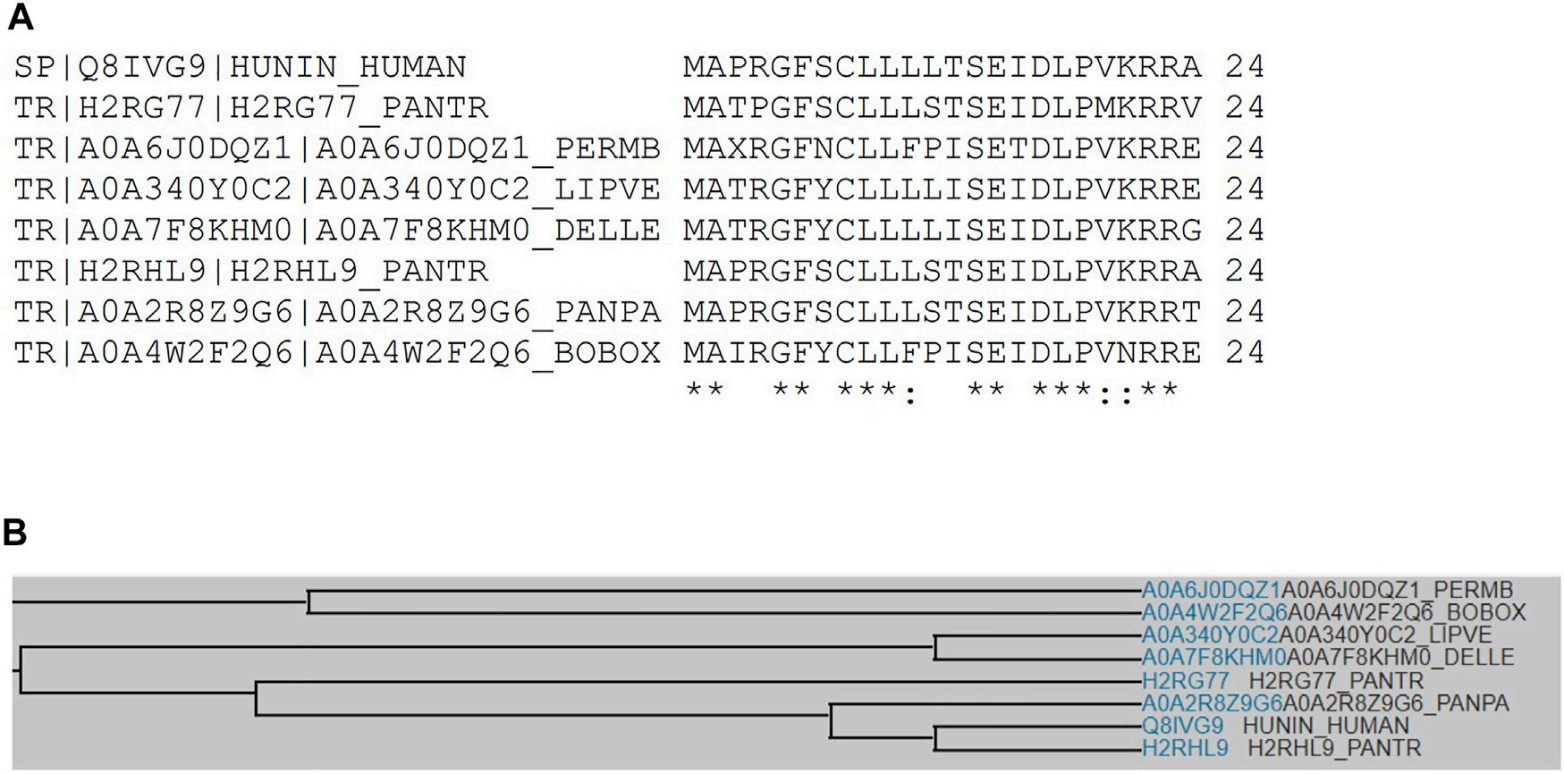
**(A)** Multiple sequence alignment analyses show that human HN shares sequence identity or similarity to Chimpanzee, Prairie deer mouse, Yangtze river dolphin, Beluga whale, Bonobo, and hybrid cattle. **(B)** A family tree of HN among these species is presented.

Sequence analysis shows that fourteen human HN isoforms are known, which share significant amino acid similarity as depicted by multiple sequence alignment (Figures 1A,B). Human HN also shares substantial sequence identity or similarity to pan troglodytes (Chimpanzee), peromyscus maniculatus bairdii (Prairie deer mouse), lipotes vexillifer (Yangtze river dolphin), delphinapterus leucas (Beluga whale), pan paniscus (Bonobo), and bos indicus x bos taurus (Hybrid cattle) (Figures 2A,B). These data suggest that HN has a conserved structure and shared functions among species.

**FIGURE 3:**
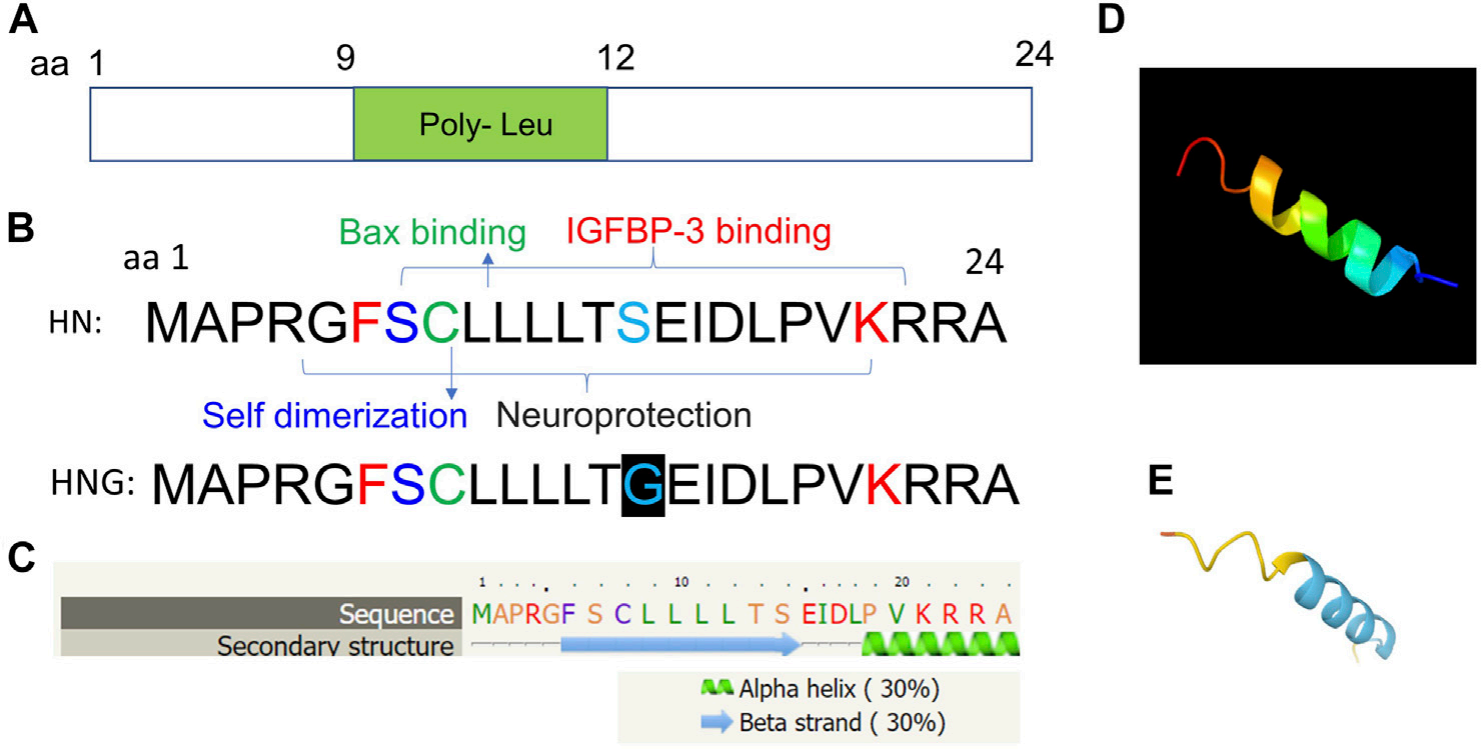
Predicted molecular structural analyses of HN. **(A)** HN is a small peptide of 24 amino acid residue and is comprised of a poly-leu motif (amino acid residues 9–12). **(B)** Key residues for BAX binding (amino acid residue 8), IGFBP3 binding (amino acid residues 6–21), and self-dimerization (amino acid residue 7) are shown. The full sequence of HN and its analogue HNG (S14G) with substitution of serine at amino acid residue 14 to glycine are shown. **(C)** Secondary structure predicts characteristics of a beta-sheet domains (amino acid residues 6–14) and an alpha-helix (amino acid residues 19–24) based on bioinformatic analysis. **(D, E)** 3D structure analyses are performed using the Phyre2 and (http://www.sbg.bio.ic.ac.uk/phyre2/) **(D)**, and AlphaFold web portals (https://alphafold.ebi.ac.uk/) **(E)**.

Molecular structure analysis showed that it contains key residues for BAX binding (amino acid residue 8), IGFBP3 binding (amino acid residues 6–21), and self-dimerization (amino acid residue 7). In addition, it is comprised of a poly-leu motif (amino acid residues 9–12) (Figures 3A,B). Secondary structure predicts characteristics of a beta-sheet domains (amino acid residues 6–14) and an alpha-helix (amino acid residues 19–24) based on bioinformatic analysis (Figure 3C). Further, the 3D structure of HN is also predicted using the Phyre2 (Kelley et al., 2015) (Figure 3D), and AlphaFold web-based portals (Jumper et al., 2021) (Figure 3E). Its neuroprotective effect is mapped to amino acid residue 3–19 with a potency determinant at amino acid residue 14 (S14G, named as HNG). HNG was reported to have a reduced helical propensity and a higher conformational flexibility when compared with wild type HN (Rojo-Dominguez et al., 2007). A HN specific binding site on the surface of Bid, was mapped to the BH3 domain that is involved with the regulation of cytoprotective activity of a cell (Choi et al., 2007).

Gene expression studies show that *HN* is abundantly expressed in the heart, kidney, testis, skeletal muscles, and at less levels in brain and liver by the measurement of quantitative RT-PCR (Bodzioch et al., 2009). In addition, most of the HN-like protein encoding genes are also expressed in testis, whilst *MTRNR2L1*, *MTRNR2L8*, and *MTRNR2L9* are also highly expressed in heart, kidney, and testis (Bodzioch et al., 2009). Additional research has shown that *HN* is expressed in the muscles of patients with chronic progressive external ophthalmoplegia (Kin et al., 2006). It remains to be determined how the expression of *HN* and *HN*-like genes are regulated differentially in different cell and tissue types.

**FIGURE 4:**
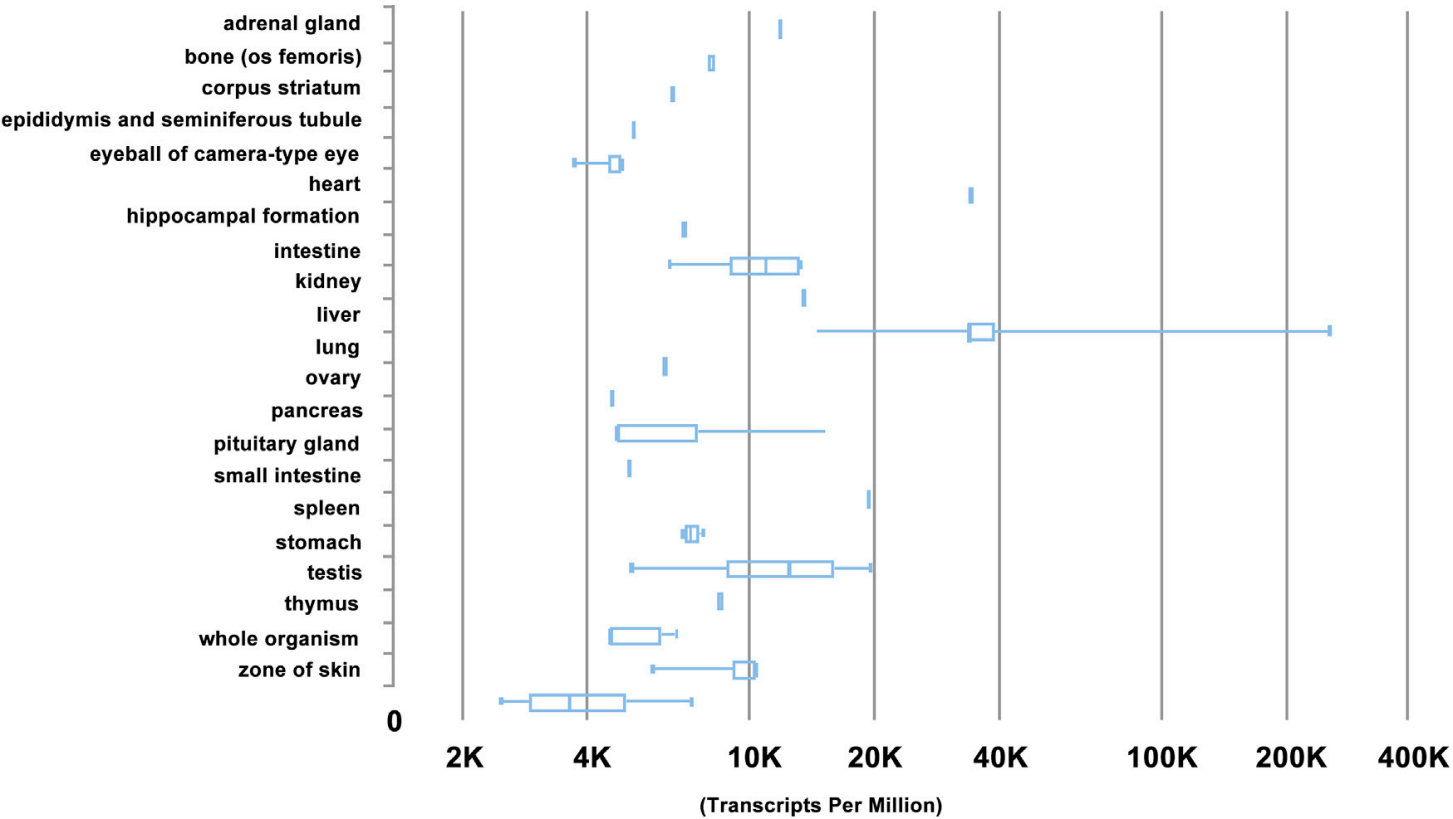
Gene expression profile of HN by RNA-Seq CAGE (Cap Analysis of Gene Expression) analysis of mouse tissue**s** in RIKEN FANTOM5 project. Note that HN is most abundantly in mouse tissue types of liver, heart, small intestine, and testis, based on https:// www.ebi.ac.uk/gxa/experiments/.

**FIGURE 5:**
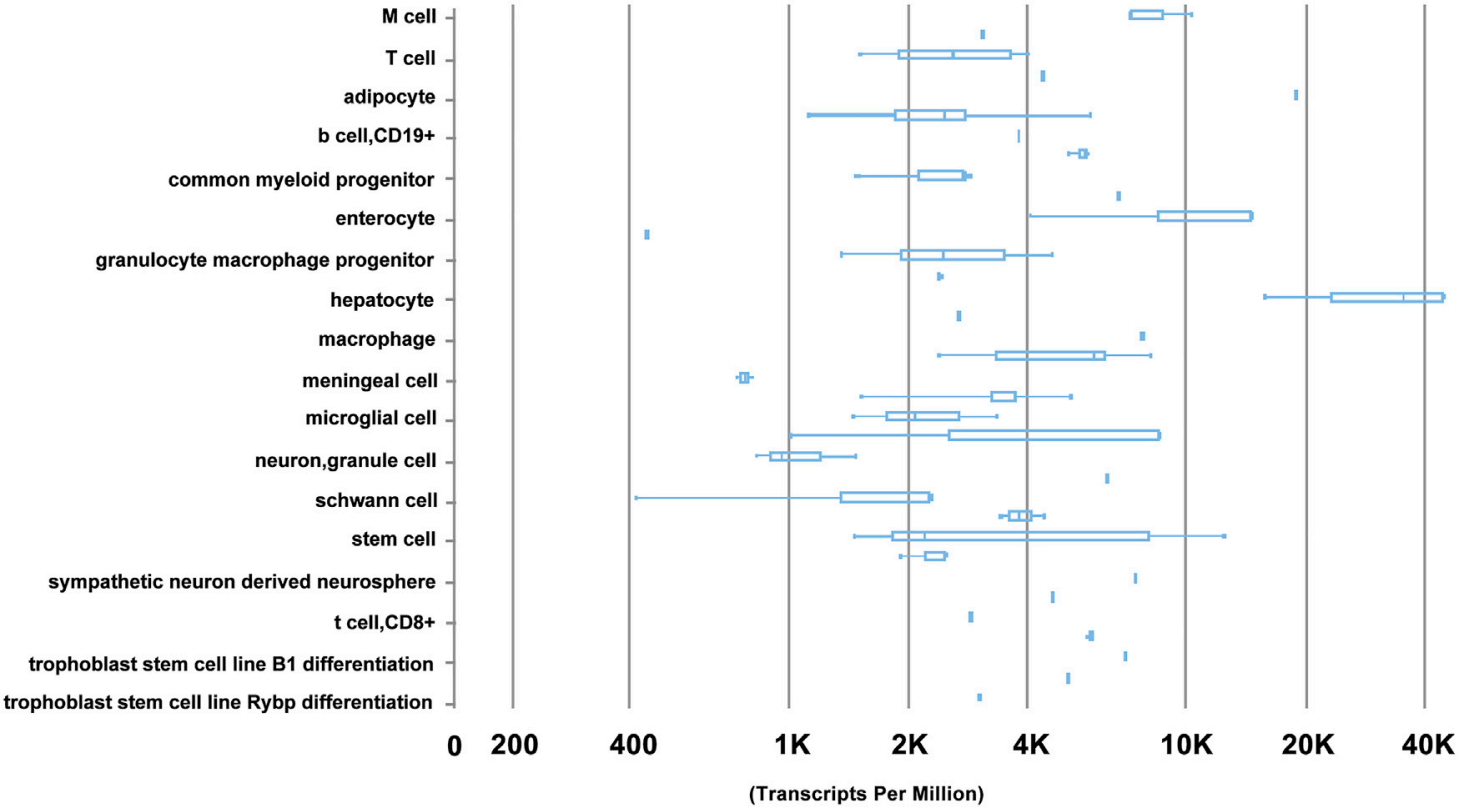
Gene expression profile of HN by RNA-Seq CAGE (Cap Analysis of Gene Expression) analysis of mouse cells in RIKEN FANTOM5 project. Note that HN is most abundantly in mouse cell types of hepatocytes, adipocytes, and enterocytes, based on https:// www.ebi.ac.uk/gxa/experiments/.

Gene expression of HN was also analysed using RNA-Seq CAGE (Cap Analysis of Gene Expression) bioinformatics in RIKEN FANTOM5. It was revealed that *HN* was most abundantly expressed in mouse tissue types of liver, heart, small intestine, and testis (Figure 4), and in mouse cell types of hepatocytes, adipocytes, and enterocytes (Figure 5). These results are consistent with RT-PCR analysis (Bodzioch et al., 2009).

In addition to mitochondrial-encoded HN, 13 human HN peptides were predicted to be encoded by MT-RNR2-like nuclear genes in various chromosomes by bioinformatic analysis (Bodzioch et al., 2009). Compared to *MT-RNR2* gene, several residues appear to be conserved including Ser7, Cys8, Leu9, and Ser14, while variable residues are also present including Pro3, Leu12, and Thr13 (Bodzioch et al., 2009). It is predicted that these various isoforms might contribute to differential neuroprotective effects and receptor binding, but their individual roles remain to be investigated. However, single nucleotide polymorphisms of 13 HN nuclear genes have no associations with coronary artery disease (Eltermaa et al., 2019).

## 3 Synthetic Analogues of HN

A synthetic derivate/analogue of HN, called HN-S14G (HNG), was designed with amino acid substitution of serine at amino acid residue 14 to glycine (Hashimoto et al., 2001b), which demonstrated more potent effects than wild type HN on the reduction of intracellular reactive oxygen species (ROS), preservation of mitochondrial membrane potential and structure with cardioprotective activity *in vivo* (Klein et al., 2013; Minasyan et al., 2017). Consistently, HNG also showed reduced damage to the mitochondrial membrane and DNA integrity, and attenuation of the reaction of oxidative stress and the activity of caspase-3 in sperm (Yang et al., 2019). Pre-treatment using HNG also showed protective effects on epidermal stem cells (ESC) against ultraviolet (UV)-B-induced cytotoxicity (Wang et al., 2019). Moreover, HNG treatment appears to ameliorate the reduction of mitochondrial membrane potential mediated by UV-B and could preserve ESC viability via a Wnt/beta-catenin signalling cascade (Wang et al., 2019). A HN derivative, colivelin was generated as a hybrid peptide containing activity-dependent neurotrophic factor (ADNF) C-terminally fused to a shorter 17-amino acid form, AGA-(C8R)HNG17 which was able to restore cognitive function in both an AD mouse model, and in AD patients involving JAK2/STAT3 signalling (Chiba et al., 2005; Arisaka et al., 2008). Further, colivelin also protect ischemic brain injury *via* rescuing ischemic neuronal death and JAK/STAT3 signalling, suggestive of a potential therapy in ischemic stroke (Zhao et al., 2019).

HNGF6A, an analogue of HN, was found to reduce atherosclerotic plaque size in the proximal aorta of ApoE-deficient mice via preserving the expression of endothelial nitric oxide synthase in aorta, and preventing endothelial dysfunction (Oh et al., 2011). More recently, novel synthetic analogues of HN, called HUJInin and c(D-Ser14-HN) were found to resist oxygen-glucose-deprivation and reoxygenation -induced neurotoxicity via modulation of Erk 1/2 and AKT phosphorylation and mitochondrial functions (Gilon et al., 2020). Consistently, HN related peptides also inhibited oxidant-induced senescence and improved mitochondrial respiration function, with an increased level of transcription factor A as well as DNA copy number of mitochondrial in human retinal pigment epithelial cells (Sreekumar et al., 2016).

## 4 Mechanisms of HN as an Intercellular Protein

At the molecular level, structural studies indicate that HN was able to undergo self-dimerization (Yamagishi et al., 2003). The protein level and stability of HN is mediated by tripartite motif containing 11 (TRIM11), an E3 ubiquitin-protein ligase, and loss of TRIM11 resulted in decreased level of HN (Niikura et al., 2003). Further, this process was mediated by proteasome inhibitor, suggesting it is regulated through ubiquitin-proteasome pathway degradation (Niikura et al., 2003).

Intracellularly, HN binds Bcl2-associated X protein (BAX), Bim and Bid and IGFBP3 to regulate cell apoptosis and survival (Guo et al., 2003; Zhai et al., 2005; Lue et al., 2010; Njomen et al., 2015). HN interacts with cytosolic Bax and cBid to inhibit their translocation to the mitochondrial membrane. In addition, HN was found to bind the membrane bound Bax and tBid, stopping cytosolic Bax oligomerization and translocation to the mitochondrial membrane. Thus, HN impedes Bax pore formation in mitochondrial outer membrane, leading to suppression of cytochrome c release and mitochondria-dependent apoptosis (Ma and Liu, 2018). Similarly, HN inhibits BimEL-induced oligomerization of Bak in mitochondria, leading to an anti-apoptotic effect on the cell (Luciano et al., 2005). In testicular germ cells, it was found to bind intracellular IGFBP3, a proapoptotic factor, which regulates its interaction with importin-beta1, and thus mediates IGFBP3-induced cell activity (Lue et al., 2010; Njomen et al., 2015). Interestingly, recent studies revealed further mechanistic insights into the role of HN in mitochondria-dependent effect. HN was found to regulate fibrillation of sequestration of pro-apoptotic BCL-2 proteins, BAX and BID into beta-sheets, which leads to inhibition of mitochondrial outer membrane translocation and oligomerization (Ma and Liu, 2018; Morris et al., 2020). However, HN does not form fibres with anti-apoptotic BCL-xL proteins (Morris et al., 2020).

## 5 Signalling of HN as a Secreted Protein

In addition to its role in mediating intracellular mitochondrial status, HN can be released as a secreted factor for autocrine signalling back to its cell surface receptor, leading to additional cytoprotective effects. HN was found to be an extracellularly secreted factor whereby two amino acid structures, Leu9-Leu11, and Pro19-Va120, appear to be essential for the secretion of HN peptide (Yamagishi et al., 2003). It was found that HN acts as a ligand for the cell surface G protein-coupled formylpeptide receptor-like 1 (FPRL1) and formyl peptide receptor-like 2 (FPRL2) (Harada et al., 2004; Ying et al., 2004). The binding of HN to FPRL1/2 was found to inhibit apoptosis signal-regulating kinase (ASK) and c-Jun N-terminal kinase (JNK) -mediated neuronal cell death (Zapala et al., 2011). More recently, it was revealed that secreted HN binds cell surface putative trimeric receptors ciliary neurotrophic factor receptor (CNTFR) alpha (CNTFR-α), gp130, and WSX-1 (or receptor for IL-27) (CNTFR-α/gp130/WSX-1), leading to the activation of intracellular signalling *via* STAT3 for neuroprotection (Hashimoto et al., 2009a; Hashimoto et al., 2013). In this study by Hashimoto et al., overexpression or siRNA-based knockdown of CNTFR-α and/or WSX-1 was found to affect HN binding to neuronal cells, and depletion of CNTFR-α or WSX-1 diminished HN-mediated cytoprotection in neurons. Interestingly, HN was also found to induce the hetero-oligomerization of CNTFR-α, WSX-1, and gp130, further indicating that the CNTFR-α/gp130/WSX-1 complex is involved in HN binding to neuronal cells (Hashimoto et al., 2009a). Interestingly, an alternatively spliced WSX-1 isoform (soluble WSX-1 or sWSX-1), which is expressed in neuronal cells was also found to convey an anti-AD activity, suggesting that CNTFR-α/soluble WSX-1/gp130 might serve as an alternative putative receptor for HN (Hashimoto et al., 2009b). Consistently, HN was shown to inhibit heat-induced germ cell apoptosis via STAT3 phosphorylation mediated through WSX-1 and gp130 (Jia et al., 2021). Further, HN binds a novel IL-6-receptor-related receptor(s) to inhibit neuronal cell death and dysfunction, and this process is involved in CNTFR-α, WSX-1, and gp130 (Matsuoka and Hashimoto, 2010). HN also binds heterotrimeric HN receptor (htHNR) to inhibit neuronal cell death caused by a familial AD-linked gene (Matsuoka, 2015). More recently, HN was found to be released in the form of exosomes from SH-SY5Y cells, which might contribute to inter-cellular or inter-tissue signalling (Wang et al., 2020).

Apart from binding to surface receptors, HN also interacts with V-set and transmembrane domain containing 2 like (VSTM2L) as a secreted protein. It was found that HN colocalizes with VSTM2L in brain regions and in cultured neuron cells *in vitro* (Rossini et al., 2011). While HN has a neuroprotective effect, VSTM2L acts as a strong antagonist of HN activity in neuron cells (Rossini et al., 2011). More recent studies have found that HN also binds extracellular IGFBP-3, a family of insulin-like growth factor I (IGF)-binding proteins, to decrease circulating IGF-I levels. In turn, IGF-I also seems to regulate HN levels (Lee et al., 2014; Xiao et al., 2016).

**FIGURE 6:**
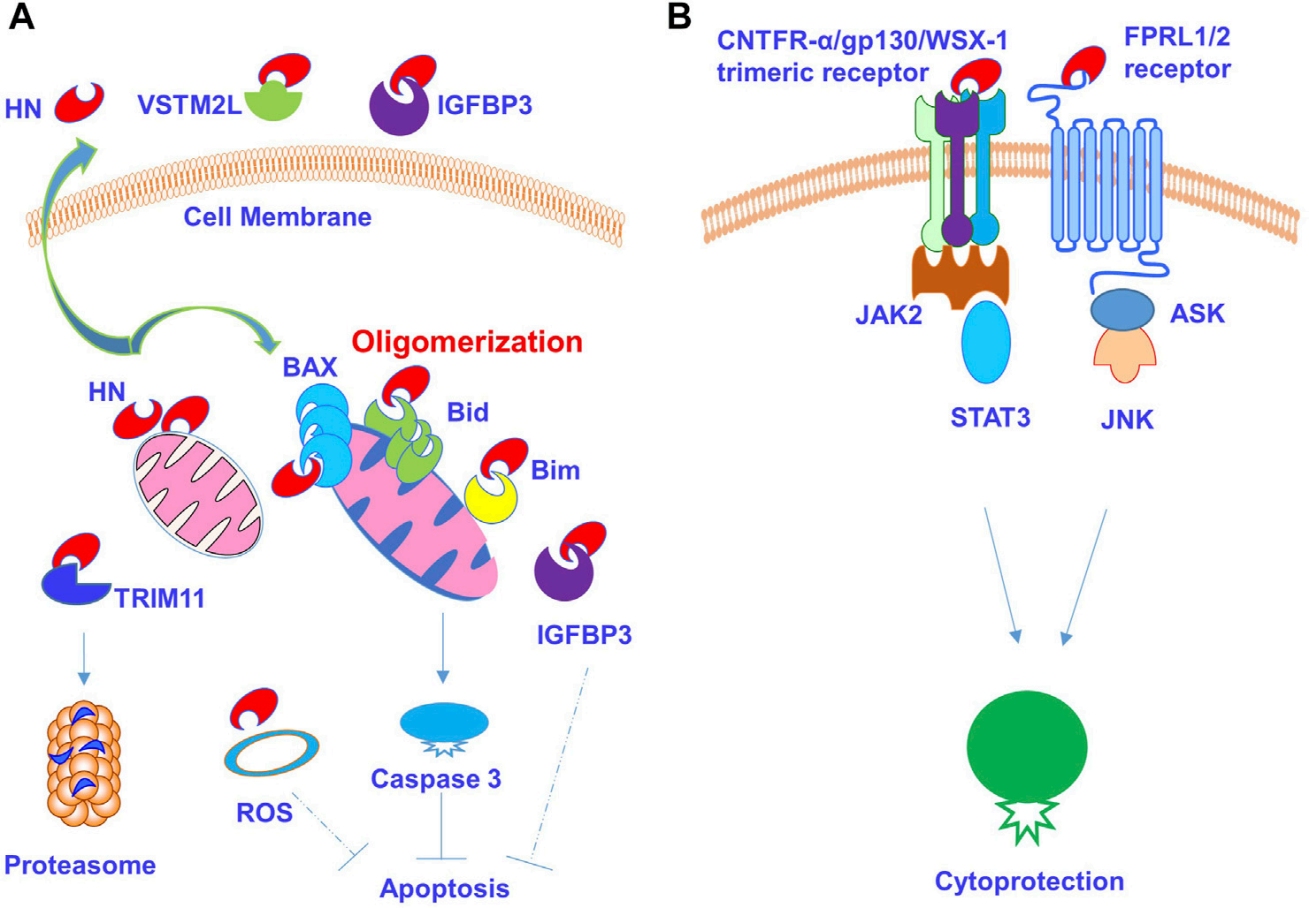
HN and its receptors signalling pathways. **(A)** HN is present intracellularly and regulate apoptotic signalling pathways and cellular activity. HN binds Bcl2-associated X protein (BAX), Bim and Bid and causes their oligomerization. HN binds intracellular IGFBP3 to mediate apoptosis. HN also diminishes intracellular ROS production. **(B)** Secreted HN binds cell surface receptors and mediates signalling pathways and cytoprotective effect. It also binds soluble extracellular proteins such as VSTM2L and IGFBP3.

A working model of NH and its binding partners is presented in Figure 6, in which HN is present intracellularly and binds to BAX, Bim, tBid, and IGFBP3 for the inhibition of apoptosis. HN has also been found to reduce ROS activity, and the level of HN protein might in turn be regulated by TRIM11 via a ubiquitin proteasome pathway (Figure 6A). HN is also secreted to bind cell surface receptors, such as G protein-coupled formyl peptide receptor-like 1 (FPRL1/FPRL2), to induce the activation of MAPK signalling pathways. Additionally, it binds CNTFR-α/gp130/WSX-1 trimeric receptors to induce the activation of JAK2/STAT3 signalling pathway, leading to cytoprotective effect (Figure 6B). In addition, HN also binds soluble extracellular proteins, such as VSTM2L and IGFBP3 to modulate cytoprotection.

## 6 The Role of HN and HN-like Proteins in Neural Disorders

HN is encoded by the *MT-RNR2* gene located within the mitochondrial genome and was first described to display a neuroprotective effect (Hashimoto et al., 2001a; Hashimoto et al., 2001b; Caricasole et al., 2002). HN is thought to suppress neuronal cell death caused by insults of AD, including amyloid-β (Aβ) peptides, and inherited AD genes, such as Swedish mutant (Hashimoto et al., 2001a; Hashimoto et al., 2001c). HN was subsequently found to act as an anti-apoptotic factor by binding and interfering with Bax (Bcl2-associated X protein; an apoptosis-inducing protein) to inhibit the translocation of Bax from the cytosol to the mitochondria, thereby actively preventing the release of apoptogenic proteins including cytochrome C to mitochondria (Guo et al., 2003). Additional studies showed that HN protected neuronal cells from Aβ1-43 and V642I- Amyloid precursor protein (AβPP)-induced cytotoxicity, which is further suggestive of its protective role against AD (Hashimoto et al., 2001b; Niikura et al., 2004). HN was found to block cytopathic effects caused by Abeta (42) in neuroblasts *via* FPRL1 receptor activation, indicating that HN might competitively inhibit Abeta (42) from access to FPRL1 (Ying et al., 2004). HN appears to protect against prion protein (PrP), PrP (118–135) fragment-induced cell death and apoptotic events in a rodent model of AD (Sponne et al., 2004). HN might inhibit neurodegeneration caused by polyQ diseases, such as dentatorubral-pallidoluysian atrophy (DRPLA), through the suppression of ASK1 and preventing the formation of polyQ aggregates (Kariya et al., 2005). HNG could attenuate Aβ (25–35)-induced neural cell injury and apoptosis by arresting mitochondrial dysfunction (Jin et al., 2010), and block the disruptive effects of Aβ 40 on the neuronal cell membrane, thereby reversing cell injury for the protection of hippocampal neurons (Li et al., 2013). HN also protected cortical neurons against AD-related okadaic acid-induced neurotoxicities, such as neuronal insults, apoptosis, and tau hyperphosphorylation (Zhao et al., 2014), and attenuated N-methyl-D-aspartate (NMDA) receptor-mediated excitotoxicity of neurological diseases by alleviating mitochondrial dysfunction, the excessive production of both ROS and nitric oxide (NO) in a rodent model (Cui et al., 2017).

More recently, HN was found to protect neuroblastoma cells against silver nanoparticles -induced neurotoxicity in neuroblastoma cells for the potential treatment of neurodegeneration (Gurunathan et al., 2019). In line with this, HN also protects calyculin A -induced neurotoxicities in cultured cortical neurons via reserving protein phosphatase 2A activity and alleviating oxidative stress in neurodegenerative diseases (Zhao et al., 2021).

Similarly, using cultured hippocampal neurons, HN was found to prevent dendritic atrophy and reduction in puncta number area for pre-synaptic marker synaptophysin (Zarate et al., 2019). It also plays a role in cognition associated with aging and post menopause (Yen et al., 2018; Zarate et al., 2019). Further understanding the protective effect and molecular mechanisms of HN on neuronal cells will help to develop HN as a promising therapeutic agent against neurodegenerative diseases.

## 7 The Role of HN and HN-Like Proteins in Skeletal Diseases

Bone homeostasis is largely regulated by the cellular activities of bone-resorbing osteoclasts and bone-forming osteoblasts. There is an emerging role of HN in skeletal diseases via the regulation of osteoclasts and osteoblasts (Zhu et al., 2020); with the cellular functions of both required to maintain a physiological balance during bone remodelling (Kular et al., 2012; Zhu et al., 2018). Research suggests that HN could be a critical regulator of osteoclastogenesis and might protect against bone disorders through the activation of AMP-activated protein kinase (AMPK) (Kang et al., 2019). AMPK inhibits receptor activator of nuclear factor-κB ligand (RANKL), and HN was found to inhibit both RANKL-induced osteoclastogenesis and the production of RANKL-induced ROS by increasing the activity of AMPK and the gene expression of *NFATc1*, *OSCAR*, *CTSK*, and *TRAP* (Kang et al., 2019). The protective function of HN was investigated for osteoblast differentiation and apoptosis (Zhu et al., 2020). HNGF6A, an analogue of HN, exerted cyto-protection from oxidative stress-induced apoptosis and promoted an osteoblast phenotype in MC3T3-E1 cells (Zhu et al., 2020). Further mechanistic studies revealed that HN promoted an osteoblast phenotype with expression of *ALP*, *BMP-2*, *OCN*, and *RUNX2* genes via modulating mitogen-activated protein kinase (MAPK) signalling pathways of p38 and JNK (Zhu et al., 2020). Interestingly, HN treatment led to decreased Circ_0001843 and increased miR-214 levels, whilst inhibition of Circ_0001843 induced the expression of miR-124, suggesting that miR-214 was direct target of Circ_0001843 (Zhu et al., 2020). However, other studies have found that miR-214 is a negative regulator of osteoblast activity and bone formation (Li et al., 2016; Sun et al., 2018; Yuan et al., 2019). The exact mechanisms of HN on osteoblast activity and bone formation will require further investigation.

Chondrocytes have been shown to play an important part in arthritis (Zhang et al., 2016; Guo et al., 2018) and arthrofibrosis (Usher et al., 2019). Interestingly, HN seems to play a protective role for chondrocytes (Eriksson et al., 2014; Zaman et al., 2019; Celvin et al., 2020). Using a DBA/1 mouse model of collagen type II induced arthritis, it was found that HNG decreased pathological scores of erythema and swelling of the joints, as well as paw histological scoring (Celvin et al., 2020), demonstrating that HNG protected from dexamethasone -induced chondrocyte apoptosis in both articular and growth plate cartilage. This study suggests that HNG complemented the therapeutic effect of glucocorticoids (GCs)-induced cell apoptosis for the treatment of chronic inflammation, and that combination therapy by HNG and GCs could be a viable treatment strategy (Celvin et al., 2020). Interestingly, HN could bind IGFBP3 in the carboxyl terminal region of the protein, which interacts with hyaluronan (HA), a key component of chondrocytes, suggesting that HN appears to mediate the cytotoxic effects of IGFBP-3 in chondrocytes through a mechanism that is involved with HA and its receptor CD44 complex (Muterspaugh et al., 2018; Dorandish et al., 2020).

Long-term use of GCs for the treatment of chronic conditions is known to impair bone growth, which is linked to the inhibition of chondrocyte function at the growth plate. The ability of HN to prevent undesirable long-term effects of GCs on bone growth was tested in mouse (Zaman et al., 2019). Results showed that HN could prevent GCs-induced bone growth impairment, chondrocyte apoptosis, and the suppression of chondrocyte proliferation (Zaman et al., 2019). Further, HN overexpression in mice protected against GC-induced growth impairment. GC treatment was found to reduce Indian Hedgehog (Hh) expression in growth plates of wild-type mice but not in HN overexpressing mice or wild-type mice treated with HN; whilst vismodegib, an Hh antagonist, was found to suppress the growth of cultured rat metatarsal bones, and this effect was also prevented by HN, indicating that HN appears to be a regulator of Hh signalling (Zaman et al., 2019). Interestingly, HNG was shown to prevent LPS-induced up-regulation of TNF-α but did not affect Dexa-mediated TNF-α levels. Similarly, HNG suppressed LPS-induced IL-6, and did not affect the anti-IL-6 effects of Dexa when used in combination (Zaman et al., 2019). These data suggest that HN and GCs could be used in combination for the treatment of chronic disease (Zaman et al., 2019).

HNG also appears to protect against chemotherapy-induced cell damage without interfering with the chemotherapy-induced suppression of cancer cells. For instance, HNG was found to prevent bortezomib-induced bone growth impairment without interfering with the desired anti-cancer effects of bortezomib (Eriksson et al., 2014). HNG has been shown to enhance cyclophosphamide (CP)-induced suppression of cancer metastases and to protect against the CP-induced suppression of male germ cells and leukocytes, whilst acting as a caloric-restrictor by suppressing IGF-1 levels (Lue et al., 2015). Similarly, HNG ameliorated temozolomide (TMZ)-induced germ cell apoptosis, white blood cell and granulocytes loss, and body weight loss, without compromising TMZ’s anti-cancer effects on medulloblastoma in severe combined immune-deficiency (SCID) mice (Jia et al., 2019).

The routes of administration of HN could include systematic intraperitoneal injection with saline (Jia et al., 2019; Zaman et al., 2019), or local injection with saline (Xu et al., 2008).

Additionally, HN exhibits potential characteristics of an oncopeptide, which would caution the use of exogenous HN to treat degenerative diseases, such as AD (Maximov et al., 2002; Moreno Ayala et al., 2020). HN appears to be involved in the progression of triple negative breast cancer (TNBC) and represents a potential therapeutic target to improve the efficacy of chemotherapy for breast cancer (Moreno Ayala et al., 2020). However, the role of HN in tumour development is incompletely understood and will require further investigation.

## 8 Summary

HN protects cells against diverse pathological conditions, including neural and skeletal diseases. HN exerts pro-apoptotic activity by binding to its BCL-2 family of proteins such as BAX to regulate mitochondrial status or by binding through extracellular FPRL1/2 receptor or trimeric receptors CNTFR-α/gp130/WSX-1. HN and its potent form HNG might serve as therapeutic agents for oxidative stress, apoptosis for neurodegenerative and skeletal diseases, and tissue regeneration. Further research, such as small animal models of spinal cord injury and osteonecrosis, combined with advanced HN delivery strategies, for example nanotherapy or bio-scaffolds, will promote the therapeutic potential of HN. The challenges and future directions for transferring the pre-clinical data on HN peptides into the clinics will require appropriate large animal models as well as the full investigation on the safety and efficacy profile of HN.
